# The dynamics of mucosal-associated invariant T cells in multiple sclerosis

**DOI:** 10.1186/s40064-016-2923-9

**Published:** 2016-08-05

**Authors:** Chie Sugimoto, Makoto Hirotani, Kazunori Yoshikiyo, Uichi Koshimizu, Rika Wakao, Takahiro Horinouchi, Yuichi Mazaki, Tsunehiko Higashi, Toshiyuki Fukazawa, Hiroyoshi Fujita, Hidenao Sasaki, Hiroshi Wakao

**Affiliations:** 1 Department of Hygiene & Cellular Preventive Medicine, Graduate School of Medicine, Hokkaido University, Sapporo, Japan; 2 Department of Neurology, Graduate School of Medicine, Hokkaido University, Sapporo, Japan; 3 Asubio Pharma Co Ltd, Kobe, Japan; 4 Pharmaceutical and Medical Device Agency (PMDA), Tokyo, Japan; 5 Department of Cellular Pharmacology, Graduate School of Medicine, Hokkaido University, Sapporo, Japan; 6 Sapporo Neurology Hospital, Sapporo, Japan

**Keywords:** Multiple sclerosis, Mucosal associated invariant T cells, FTY720, Disease-modifying therapy, Phenotyping

## Abstract

**Background:**

Multiple sclerosis (MS) is an autoimmune disease characterized by inflammatory demyelination, gliosis and axonal loss in the Central Nervous System. Although the etiology of the disease has remained enigmatic, recent studies have suggested a role of the innate-like T cells, called Mucosal Associated Invariant T cells (MAITs) in the pathophysiology. In the present study, we have analyzed the relative frequency of MAITs and the expression of the cell surface antigens in MAITs to seek a possible link to the disease.

**Results:**

There was little difference in the frequency of total MAITs between healthy donors (HDs) and untreated MS patients, whereas the latter harbored more CD8^lo/neg^ (DN) MAITs concomitant with a decrease in CD8^high^ MAITs and in CD4 MAITs compared with those in HDs. While the expression of CCR5, CCR6, CD95, CD127, and CD150 has increased in untreated subjects compared with that in HDs, CD45RO has declined in untreated subjects in both DN MAITs and CD8^hi^ MAITs. FTY720 therapy has increased the relative frequency of total MAITs in a time-dependent fashion up to 2 years. Intriguingly, FTY720 therapy for 3 years reversed the above phenotype, engendering more CD8^high^ MAITs accompanied with decreased DN MAITs. FTY720 therapy affected the cytokine production from CD4 T cells and also enhanced the relative frequency of cells producing both TNF-α and IFN-γ from MAITs, CD8 T cells, and CD4 T cells compared with that in untreated subjects.

**Conclusions:**

FTY 720 therapy enhanced the relative frequency of MAITs in MS patients in a time-dependent manner. Although the expression of CD8 in MAITs has been affected early by FTY720, longer treatment has reversed the phenotypic change. These data demonstrated that FTY720 induced dynamic change in the relative frequency and in the phenotype of MAITs in MS.

**Electronic supplementary material:**

The online version of this article (doi:10.1186/s40064-016-2923-9) contains supplementary material, which is available to authorized users.

## Background

Multiple sclerosis (MS) is a primary cause of neurological disability in young adults and has been regarded as an autoimmune disease characterized by inflammatory demyelination, gliosis and axonal loss in the central nervous system (CNS) (Compston and Coles 2008; Confavreux and Vukusic 2006). The etiology of MS has remained elusive to date; however, the involvement of auto-reactive T and/or B cells has been suspected (Huseby et al. 2012; Pröbstel et al. 2015). Implication of immune cells in the pathophysiological processes of MS have been underpinned by the fact that immunosuppresive or immunomodulatory therapies are effective in reducing the relapse number and in delaying the progression of relapsing remitting MS (Brinkmann 2009; Lim and Constantinescu 2010). Among the drugs used for MS, FTY720, an agonist of sphingosine-1-phosphate (S1P) receptors, exhibits its efficacy by inhibiting the egress of naïve and central memory T and B cells from lymph nodes and probably through secondary beneficial effects on glia in the CNS via S1P receptors (Brinkmann 2009). Oral administration of FTY720 resulted in a marked reduction in lymphocyte number (lymphopenia), in particular, in naïve and central memory T and B cells expressing chemokine receptor CCR7 being present in the secondary lymphoid organs and in peripheral blood. In contrast, effector/memory T cells without CCR7 expression showed a relative increase during FTY720 therapy (Brinkmann 2009).

Recent studies have revealed that a subset of innate-like T cells called mucosal-associated invariant T cells (MAITs) could play a role in the pathophysiology of MS. MAITs are characterized by their abundance in humans, occupying up to 10, 20, and 50 % of T cells in the intestinal lamina propria, in the peripheral blood, and in the liver, respectively (Dusseaux et al. 2011) (unpublished results). Contrary to conventional T cells, MAITs recognize vitamin B2 metabolites as antigens and promptly produce a plethora of cytokines and chemokines upon activation (Birkinshaw et al. 2014; Le Bourhis et al. 2011; Wakao et al. 2013). MAITs are believed to play a pivotal role in host defense against bacterial and fungal infection as well as in human autoimmune diseases such as inflammatory bowel disease, systemic lupus erythematosus, psoriasis, and MS (Cho et al. 2014; Miyazaki et al. 2011; Serriari et al. 2014; Teunissen et al. 2014; Treiner and Liblau 2015). In the case of MS, MAITs have been found in lesions from postmortem brain samples, implying that MAITs are implicated in the pathophysiology of MS (Illés et al. 2004; Willing et al. 2014). However, there has been debate as to the frequency of MAITs in MS. While MAITs were reported to be decreased in peripheral blood from MS patients compared with those from healthy donors (HDs), Annibali and colleagues have shown that MAITs increased along with the disease duration (Miyazaki et al. 2011; Annibali et al. 2011; Willing et al. 2014).

In the present study, we analyzed how MS and relevant drugs affected the phenotype and functions of MAITs as well as those of conventional CD4 and CD8 T cells. Our study revealed that although the phenotypic features of MAITs were affected by the disease as well as by the drugs, MAITs, in particular, had the potential to produce the cytokines relevant to fighting bacterial infections and remained quasi-intact. Furthermore, the relative frequency of MAITs between HD and untreated MS patients did not differ but it increased upon FTY720 treatment in a time-dependent manner.

## Methods

### Patients

Venous blood samples were obtained from HDs and from relapsing-remitting MS patients presenting to Sapporo Neurology Clinics and Hokkaido University Hospital, with informed consent (Table 1). The patients were either disease-modifying treatment free (hereafter referred as untreated) (14 females and 3 males; mean age ± SD, 38.4 ± 12.7 years), interferon (IFN) β1a- or b-treated (10 females and 3 males; mean age ± SD, 40.6 ± 8.6 years), or FTY720-treated (4 females and 3 males; mean age ± SD, 34.1 ± 8.4 years). Among the patients who started on FTY720 therapy in 2012 at Hokkaido University Hospital, seven patients were, in general, followed before and at 3, 12, 24 and 36 months after initiation of FTY720 (0.5 mg per day). MS was diagnosed based on the 2010 revised McDonald criteria for MS (Polman et al. 2011). The samples obtained from 16 HDs (13 females and 3 males; mean age ± SD, 38.5 ± 11.4 years) were used for phenotyping and functional assays, and those from 9 HDs (6 females and 3 males; mean age ± SD, 30.9 ± 7.7 years) were used for absolute blood cell counting. For extensive analysis of MAITs, two untreated subjects were omitted due to the paucity of MAITs (less than 0.6 %). Therefore, samples from15 untreated were used for the further analysis. In the analysis evaluating the effects of FTY720 on MAIT cell frequency and on expression of the cell surface antigens, some samples were missing due to the unavailability (see Additional file 1: Table S1).

**Table 1:** Subjects enrolled in this study

Criteria	Number	Sex (F:M)	Age		EDSS^a^	
			Median	Mean ± SD	Median	Mean ± SD
HD	16	13:3	36	38.4 ± 11.4	NA^b^	NA
Untreated (UT)	17	14:3	38	38.4 ± 12.7	1.5	1.9 ± 1.7
Follow-up after FTY treatment	7	4:3	37	34.1 ± 8.4	2	1.9 ± 1.5
IFNβ treated	12	9:3	41	41.9 ± 8.4	2	2.0 ± 1.0

^a^Expanded Disability Status Scale

^b^ *NA* not applicable

### FACS analysis

Peripheral blood mononuclear cells (PBMCs) from untreated, IFNβ-treated, FTY720-treated subjects, and HDs were prepared using a Ficoll gradient and subjected to 8-color fluorescence-activated cell sorting (FACS) analysis, as described previously (Sugimoto et al. 2015). Cell surface antigen expression was analyzed with the indicated phycoerythrin (PE)-labeled anti-human antibodies within Brilliant Violet 421-labeled CD3 (UCHT1, Biolegend, Tokyo, Japan), allophycocyanin (APC)-labeled CD161 (HP-3G10, Biolegend), and fluorescein isothiocyanate (FITC)-labeled anti-Vα7.2 (3C10, Biolegend). The reaction mixture also contained Brilliant Violet 605-labeled CD4 (RPA-T4, BD Biosciences), APC/Cy7-labeled CD8 (SK1, Biolegend), and PE/Cy7-labeled CD45RO (UCHL1, BD Biosciences, Tokyo, Japan). A complete list of PE-labeled cell surface antigens used is found in Additional file 2: Table S2.

Intracellular cytokine assays were performed as follows. Cryopreserved PBMCs were thawed and treated with DNase, then incubated for 3 h at 37 °C in 5 % CO_2_ to restore the cells. Cells were then stimulated with phorbol 12-myristate 13-acetate (10 ng/ml) and ionomycin (1 μg/ml) (both from Wako Pure Chemicals, Osaka, Japan) in the presence of 5 µg/ml each of brefeldin A (Cell Signaling Technology, Tokyo, Japan) and monensin (Wako Pure Chemicals) for 5 h at 37 °C in 5 % CO_2_. The cultured cells were stained with antibodies against PerCP/Cy5.5-TCR Vα7.2 (3C10, Biolegend), PE-Cy7-CD3 (SK7, BD Biosciences), PE-CF594-CD4 (RPA-T4, BD Biosciences), APC/H7-CD8 (SK-1, BD Biosciences), and Brilliant Violet 421-CD161 (HP-3G10, Biolegend) and permeabilized with BD Cytofix/Cytoperm solution (BD Biosciences) according to the manufacturer’s instructions. Intracellular cytokines were stained with antibodies against Alexa Fluor 488-IFNγ (B27, BD Biosciences), PE-tumor necrosis factor (TNF) α (Mab11, Biolegend), Alexa Fluor 647-granulocyte/macrophage-colony stimulating factor (GM-CSF) (BVD2-21C11, BD Biosciences), and Brilliant Violet 510-IL-17A (N49-653, BD Biosciences). Stained cells were acquired using a BD ARIA II (BD Biosciences) and analyzed using FlowJo version 9.9 (FlowJo, LLC) and SPICE version 5.35 (http://exon.niaid.nih.gov/spice, National Institute of Allergy & Infectious Diseases, NIH) (Roederer et al. 2011).

### Absolute cell counting

EDTA-anticoagulated whole blood was stained with antibodies against FITC-CD45, Brilliant Violet 421-CD3, Brilliant Violet-CD4, APC/H7-CD8, APC-CD161, and PerCP/Cy5.5-TCR Vα7.2, and lysed using 1X BD FACS lysing solution (BD Biosciences). Absolute cell numbers in each cell population were analyzed using a MACSQuant analyzer (Miltenyi Biotec) with volumetric acquisition.

### Statistics

Statistical analyses of FACS data were performed using GraphPad Prism (ver. 6). The significance of differences in the expression of cell surface antigens was evaluated using the Mann–Whitney *U* test or Kruskal–Wallis H test with Dunn’s multiple comparison test. *P* values <0.05 were considered to indicate statistical significance. Change in the relative frequency of MAITs upon FTY720 treatment was evaluated with Friedman’s test. Two-way AVONA was used for evaluating the difference among HDs, untreated- and FTY720-treated subjects in terms of the cytokine production profile from MAITs, CD4 T cells, and CD8 T cells and *P* values were corrected with Holm-Sidak test.

For the cytokine production profiling analysis, partial permutation test has been employed with SPICE for the pie chart (Roederer et al. 2011).

## Results

### Relative frequency of MAITs in MS

**Fig. 1:**
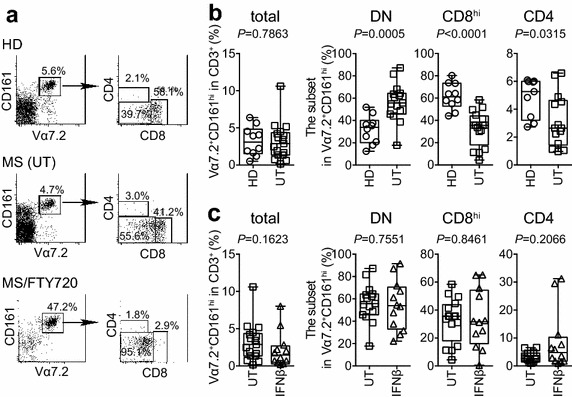
CD8 MAITs consist of CD8^hi^ and CD8^hi/lo^ (DN) MAITs cells. **a** A representative FACS profile of mucosal-associated invariant T cells (MAITs) from a healthy donor (HD), a disease-modifying treatment free (untreated:UT) subject, and an FTY720-treated MS subject (MS/FTY720). MAITs are identified as cells harboring Vα7.2 and CD161 in CD3^+^ T cells. Vα7.2^+^ CD161^hi^ cells were gated, and the expression levels of CD4 and CD8 were analyzed. The *number* in the figure shows the percentage of the indicated populations. **b** Difference in the relative frequency of MAIT cell subsets between HDs and untreated subjects. Relative frequency of total MAITs (Vα7.2^+^ CD161^hi^ cells in the total CD3^+^ cells) from HDs (n = 10) and that from untreated subjects (UT, n = 15) are plotted (total). Relative frequency of DN MAITs, CD8^hi^ MAITs, and CD4 MAITs in total MAITs are plotted (DN, CD8^hi^, and CD4, respectively). **c** The effect of INFβ on the frequency of MAITs. Relative frequency of total MAITs and that of the MAIT cell subset in total MAITs from UT (n = 15) and from INFβ-treated subjects (n = 12) are plotted (total, DN, CD8^hi^, and CD4, respectively). **b**, **c** All data are presented as *horizontal lines*: median; *boxes*: 25th percentile and 75th percentile; *whiskers*: minimum and maximum. *P* values after Mann–Whitney *U* test are shown

**Fig. 2:**
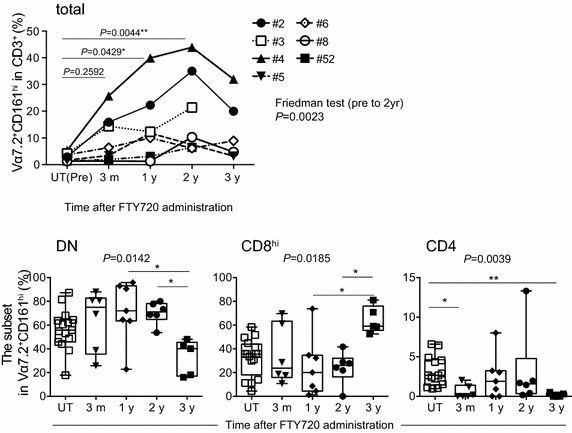
Effects of FTY720 on MAITs. *Upper panel* The frequency of MAITs post FTY720 treatment. The frequency of MAITs (Vα7.2^+^ CD161^hi^ cells) relative to total T cells (CD3^+^ cells) in the blood from subjects before (UT(pre)) and after FTY720-treatment (3 months, 1, 2 and 3 years; n = 7) is shown. Each individual was followed up to 3 years. The data are analyzed with Friedman test (from UT (Pre) to 2 years) with Dunn’s multiple comparison test for all the possible combinations. Groups showing a difference are indicated with an *asterisk* (*P < 0.05; **P < 0.01).* Lower panel*: The effect of FTY720 on the subset frequency of MAITs. The percentage of DN, CD8^hi^, and CD4 MAITs relative to total MAITs in blood from untreated subjects (UT, n = 15) and FTY720-treated (n = 7) subjects are shown. 3 months, 1, 2, and 3 years; subjects received FTY720 for 3 months, 1, 2, and 3 years, respectively. Data are analyzed with Kruskal–Wallis test with Dunn’s multiple comparison test for all the possible combinations. Groups showing a difference are indicated with an *asterisk* (*P < 0.05; **P < 0.01). All data are presented as *horizontal lines*: median; *boxes*: 25th percentile and 75th percentile; *whiskers*: minimum and maximum

MAITs are mainly composed of CD8 cells or double negative cells harboring neither CD8 nor CD4, and of few CD4 cells (Le Bourhis et al. 2011). To assess a possible implication of MAITs in MS, MAITs (Vα7.2^+^CD161^hi^) were analyzed for their expression of CD8 and CD4. We first examined the effects of the disease and of the drugs for MS on these subsets. While there was no or little difference in the relative frequency of MAITs between HD and untreated subjects, CD8^hi^ MAITs were significantly decreased in untreated subjects compared with those in HDs, resulting in more CD8^lo/neg^ MAITs (hereafter referred as double negative (DN) MAITs) in MS (Fig. 1a, b). Such depletion of CD8^hi^ cells was also observed in IFNβ- and FTY720-treated subjects (data not shown, and Fig. 1a). In marked contrast, no such change was observed in conventional CD8 T cells irrespective of the disease and the drug treatment (data not shown). Although there was no significant difference in the frequency of conventional CD4 and CD8 T cells between HD and untreated subjects, and between untreated and IFNβ-treated subjects, FTY720 induced a decline in the frequency of CD4 T cells concomitant with an increase in CD8 T cells compared with that in untreated subjects (Additional file 3: Figure S1). Then, we assessed the relative frequency of total MAITs (Vα7.2^+^CD161^hi^ cells) in T cells (CD3^+^ cells), and of CD8^hi^ MAITs, DN MAITs, and CD4 MAITs relative to total MAITs between untreated and IFNβ-treated subjects. The relative frequency of MAITs did not differ between the groups (Fig. 1c). Furthermore, IFNβ had little effect on the frequency of DN, CD8^hi^, and CD4 MAITs (Fig. 1c). We longitudinally examined the effects of FTY720 on the frequency of MAITs. FTY720 treatment for 3 months (FTY-3 m), 1 year (FTY-1y) and 2 years (FTY-2y) significantly enhanced the relative frequency of MAITs compared with that in the pre-treatment state (Fig. 2, total). Although some patients continued to increase the frequency after 1 year, others started to show a decline (Fig. 2, total). While there was no significant difference in the frequency of DN MAITs between untreated and FTY-3 m groups, it declined in subjects treated for 3 years (FTY-3y) compared with that in FTY-1y and -2y (Fig. 2, DN). Of note was that after 3 years’ treatment, the relative frequency of DN MAITs almost recovered to the level seen in HDs (median; 40.2 vs. 33.9 %, Figs. 1b, 2). The same regimen also resulted in the recovery of CD8^hi^ MAITs to a degree observed in HDs (median; 59.1 vs. 61.1 %, Figs. 1b, 2). Although FTY-3 m resulted in a significant decrease in the frequency of CD4 MAITs relative to untreated subjects, 3 years’ intake did not restore their frequency as observed in CD8^hi^ MAITs (Figs. 1b, 2).

### DN MAITs are characteristic in MS

Having established that most MAITs have decreased CD8 expression in untreated and FTY720-treated subjects compared with that in HDs, we next addressed whether DN MAITs harbored a different profile of cell surface antigen expression using HD PBMCs. Analysis with an array of antigens (Additional file 2: Table S2) has uncovered that co-stimulatory molecules CD27, CD28, and CD278 (ICOS), and an integrin family member, CD49d, exhibited decreased expression in these cells relative to that in CD8^hi^ MAITs, whereas expression of a multi-drug efflux pump, CD243, showed the opposite phenotype (Table 2 and Additional file 4: Figure S2A). On the contrary, there was no difference in expression of CCR5, CCR6, a memory marker CD45RO, CD95, CD218a [IL-18 receptor α (IL-18Rα)], and CD127 [IL-7 receptor α (IL-7Rα)] between CD8^hi^ MAITs and DN MAITs (Additional file 4: Figure S2B). Comparison among the different subsets of MAITs also revealed differences in expression. The expression of CCR4, CD95, and ICOS in CD4 MAITs showed an increase compared with that in CD8^hi^ MAITs and in DN MAITs (Table 2 and Additional file 4: Figure S2C). Conversely, the expression of an inflammatory chemokine receptor, CCR5, a dipeptidase responsible for processing several chemokines and cytokines, CD26, and a signaling lymphocyte associated molecule (SLAM) family member, CD244 declined in CD4 MAITs relative to that in CD8^hi^ MAITs and DN MAITs (Table 2 and Additional file 4: Figure S2C). Furthermore, CD27 and CD49d expression in CD8^hi^ MAITs was higher than that in DN MAITs (Table 2 and Additional file 4: Figure S2C). The expression level of CD127 (IL-7Rα) and CD279 (PD1) in CD4 MAITs increased relative to that in DN MAITs (Table 2 and Additional file 4: Figure S2C), whereas there was no difference in the expression of CCR6, CD28, CD45RA, CD107a, CD150, CD218a (IL-18Rα), NKG2D, and CD45RO (Additional file 4: Figure S2D).

**Table 2:** Cell surface antigen expression in Vα7.2^+^CD161^hi^ MAIT cells from HD

	DN	CD8^hi^	CD4
CCR4	–	–	+
CCR5	++	++	+
CCR6	+	+	+
CD26	++	++	++
CD27	+	+	+
CD28	++	++	++
CD45RA	+	+	+
CD49d	+	++	+
CD95	+	+	++
CD107a	+	+	–
CD127	+	++	++
CD150	+	+	+
CD244	+	++	–
CD279	–	+	+
ICOS	+	+	+
IL18Rα	+	+	+
NKG2D	+	+	–
CD45RO	++	++	++

− No significant expression

+ Median MFI <1000

++ Median MFI 1000>

### Profile of cell surface antigens in MAITs reflected the disease status

**Fig. 3:**
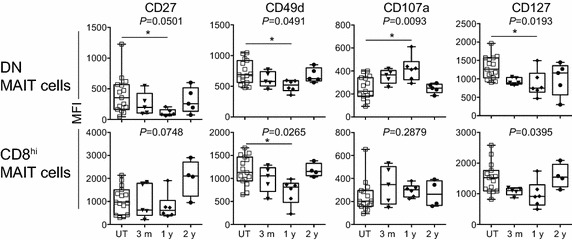
Effects of FTY720 on the cell surface antigen expression in MAITs. The mean fluorescence intensity (MFI) for the indicated cell surface antigens in DN MAITs and in CD8^hi^ MAITs from untreated subjects (n = 15), and subjects treated with FTY720 for 3 months (n = 5), 1 year (n = 6), and 2 years (n = 5), are plotted. 3 m, 1 y, and 2 y; subjects treated with FTY720 for 3 months, 1 and 2 years, respectively. Data are analyzed with Kruskal–Wallis test with Dunn’s multiple comparison test for all the possible combinations. Groups showing a statistical significance are indicated with *asterisk* (*P < 0.05). Data are presented as *horizontal lines*: median; *boxes*: 25th percentile and 75th percentile; *whiskers*: minimum and maximum

We extended our study to examine whether there were any markers in MAITs that could distinguish diseases states using an array of antigens (Additional file 2: Table S2). The expression of CCR5, CCR6, CD95, CD127, and CD150, increased in untreated subjects compared with that in HDs in both CD8^hi^ and DN MAITs ( Table 3 and Additional file 5: Figure S3A). Similarly, CD45RO decreased in untreated subjects compared with that in HDs in both CD8^hi^ and DN MAITs (Table 3 and Additional file 5: Figure S3A). We then analyzed the effects of the drugs for MS on the expression of these markers. IFNβ treatment resulted in a decrease in the expression of CCR6 and CD49d relative to that in untreated subjects, whereas the expression of ICOS was augmented in both CD8^hi^ and DN MAITs (Table 3 and Additional file 5: Figure S3B). Of note, a NK receptor, NKG2D, showed a decline only in DN MAITs. In CD8^hi^ MAITs, a decline of CD28 and CD127 expression was observed, whereas level of a degranulation marker, CD107a, and CD279 (PD-1) increased (Table 3 and Additional file 5: Figure S3B). We then assessed the effects of FTY720 on expression of the cell surface antigen in DN MAITs and CD8^hi^ MAITs. FTY-1y resulted in a decrease in the expression of CD49d and CD127 compared with those in untreated subjects in both CD8^hi^ and DN MAITs (Fig. 3). Of note, the same regimen led to a decline in CD27 expression concomitant with an increase in CD107a in DN MAITs, as observed in the terminal differentiation of effector cells (Fig. 3) (Brenchley et al. 2003). While FTY-2y resulted in an increase in CCR5 expression in CD8^hi^ MAITs compared with FTY-1y, FTY720 did not affect the expression of CD26, CD28, CD45RA, CD69, CD150, and CD243 in MAITs (Additional file 6: Figure S4A). Intriguingly, FTY-2y resulted in a decline in the expression of CD45RO in DN MAITs concomitant with an increase in CD45RA in both DN and CD8^hi^ MAITs compared with that in HDs (Additional file 6: Figure S4B).

**Table 3:** Changes in the cell surface antigen expression between HD and UT, and UT and IFNβ-treated subjects

Markers	HD vs. UT				UT vs. IFNβ			
	DN		CD8^hi^		DN		CD8^hi^	
	Difference^a^	Signifiance^c^	Difference^a^	Signifiance^c^	Difference^b^	Signifiance^c^	Difference^b^	Signifiance^c^
CCR5	0.29	****	0.27	***	−0.08	ns	−0.05	ns
CCR6	0.55	***	0.73	***	−0.44	****	−0.34	***
CD28	0.05	ns	0.14	*	0.02	ns	−0.05	*
CD45RO	−0.39	**	−0.45	**	−0.09	ns	0.02	ns
CD49d	0.06	ns	0.00	ns	−0.10	*	−0.13	*
CD95	0.18	***	0.27	***	0.02	ns	0.00	ns
CD107a	0.10	ns	0.04	ns	−0.01	ns	0.16	*
CD127	0.20	**	0.09	*	0.00	ns	−0.09	*
CD150	0.33	**	0.27	*	−0.10	ns	0.02	ns
CD279	nd	–	0.00	ns	nd	–	0.22	**
ICOS	0.09	ns	0.06	ns	0.12	*	0.06	*
NKG2D	0.05	ns	0.04	ns	−0.32	*	−0.20	ns

^a^ $$\log 10\frac{{{\text{median}}\;{\text{MFI}}\;{\text{for}}\;{\text{HD}}}}{{{\text{median}}\;{\text{MFI}}\;{\text{for}}\;{\text{UT}}}}$$

^b^ $$\log 10\frac{{{\text{median }}\;{\text{MFI}}\;{\text{for}}\;{\text{UT}}}}{{{\text{median }}\;{\text{MFI}}\;{\text{for}}\;{\text{IFN}}\beta }}$$

^c^Statistical significances are shown with asterisks (*P < 0.05; **P < 0.01; ***P < 0.001) and *ns* not significant

### Lymphocytes decreased during FTY720 treatment

**Fig. 4:**
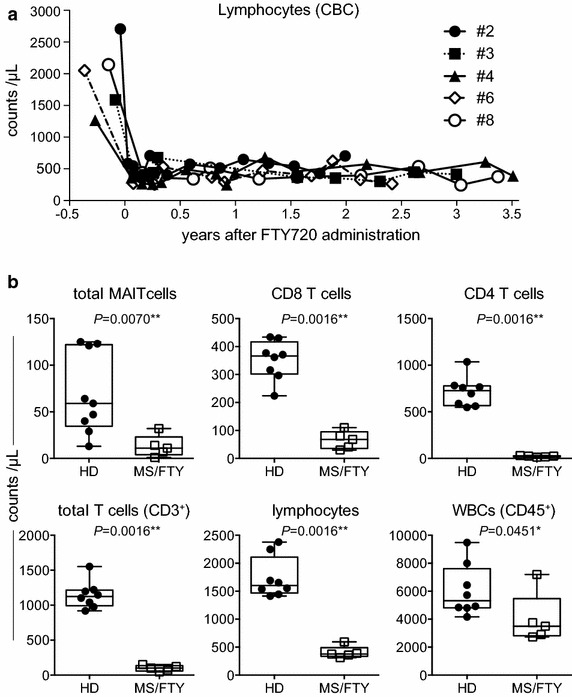
Effects of FTY720 on lymphocytes. **a** Time course of lymphocytes counts during FTY720 treatment. The lymphocyte counts in the each individual (#2, 3, 4, 6, and 8) were followed from the pre-drug to 3 years and half with a mean interval of 3 months. **b** Effect of FTY720 on blood cells. The absolute counts of the indicated subsets in the blood from HDs (n = 9) and from subjects treated with FTY720 for 3 years (n = 5) are shown. *P* < 0.05 indicates statistical significance (Mann–Whitney *U* test). All data are presented as *horizontal lines*: median; *boxes*: 25th percentile and 75th percentile; *whiskers*: minimum and maximum

We next addressed whether FTY 720 therapy led to a decrease in lymphocytes in the peripheral blood. Just 10–30 days post treatment, the absolute lymphocyte counts declined to 15–20 % of the pre-drug state in 5 patients. The absolute lymphocyte counts in each individual were kept relatively constant throughout drug treatment periods up to 3.5 years (Fig. 4a). We then measured the absolute lymphocyte number in patients treated with FTY720 for 3 years. Although the relative frequency of MAITs was twice that in HDs, the absolute number declined to one-sixth of that in HDs (data not shown and Fig. 4b). Similarly, CD8 T cell numbers represented 18.6 % of that in HDs, whereas CD4 T cell numbers were one-thirtieth of that in HDs (Fig. 4b). Notably, regardless of the fact that T cell (CD3^+^ cells) numbers declined to one-eleventh of that in HDs, the total lymphocyte number decreased to one-fifth of that in HDs. Surprisingly, white blood cell (CD45^+^ cells) numbers diminished only to two-thirds of that in HDs, indicating that FTY720 primarily affected circulating T cells, in particular CD4 T cells, in the blood (Fig. 4b).

### Effects of FTY720 on the cytokine production potential in MAITs, CD8 T cells, and in CD4 T cells

**Fig. 5:**
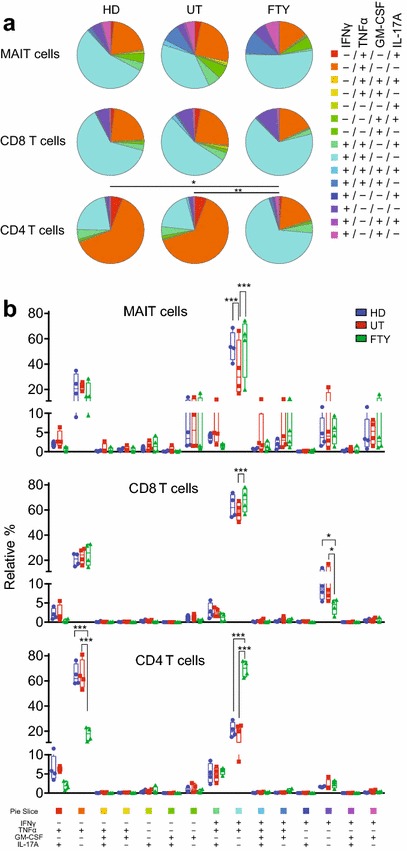
Cytokine production from the lymphocytes. **a** Comparison of the cell subsets capable of producing the cytokines. The relative percentages of cells being capable of secreting the indicated set of cytokines within MAITs, conventional CD8 and CD4 T cells from HDs (n = 4) and untreated (n = 4: UT) and FTY720-treated (treatment for 1 and 2 years, n = 4: FTY) subjects are shown as a pie chart. An *asterisk* indicates statistical significance after the partial Permutation test *P < 0.05; **P < 0.01). **b** Frequency of cells producing a different set of cytokines. The relative percentage of cells producing the indicated cytokine combination from HD (n = 4), untreated (n = 4: UT), and FTY720-treated (treatment for 1 and 2 years, n = 4:FTY) subjects is shown. The statistical significances are analyzed by Two-way ANOVA and *P* values are corrected with Holm-Sidak test. An *asterisk* indicates the groups exhibiting a statistical difference (*P < 0.05; ***P < 0.001). All data are presented as *horizontal lines*: median; *whiskers*: minimum and maximum

Given that FTY720 treatment altered the cytokine production profile in CD4 T cells (Song et al. 2014), the potential to produce IFNγ, TNFα, and IL-17A from MAITs, CD8, and CD4 T cells was assessed (Fig. 5). Besides these cytokines, the production of GM-CSF was also measured, because GM-CSF is a hallmark of MAITs and has been suggested to be pathogenic in MS (Croxford et al. 2015; Cui et al. 2015; Rahimpour et al. 2015; Wakao et al. 2013) (Additional file 7: Figure S5). SPICE analysis has revealed a difference in the cytokine production profile in CD4 T cells between and HD and FTY720-treated groups, and between untreated and FTY720-treated groups (Fig. 5a). However, no or little difference was observed in MAITs and CD8 T cells (Fig. 5a). When two way ANOVA was performed for all the possible pairs among HD, untreated, and FTY720-treated groups in terms of cytokine production profile, we observed a decline in the relative frequency of cells producing both IFNγ and TNFα in MAITs from untreated group relative to that in HD (Fig. 5b). Intriguingly, FTY720 treatment enhanced the relative frequency of these cells in MAITs, CD8 and CD4 T cells compared with that in untreated group. Similarly, CD4 T cells harbored more double producer cells upon FTY720 treatment than those in HD (Fig. 5b). In marked contrast, FTY720 treatment decreased the relative frequency of TNFα single producer cells from CD4 T cells compared with that in HD and in untreated groups (Fig. 5b). Similarly, the same regimen resulted in a decline in the frequency of IFNγ single producer cells from CD8 T cells (Fig. 5b). Although the number of the subjects was quite limited (n = 4, per group), the cytokine production test revealed the effects of the disease and of FTY720 on MAITs, CD4, and CD8 T cells.

## Discussion

It has been proposed that MAITs are implicated in the pathophysiology of MS, although the phenotype and function of MAITs in MS have not been explored extensively (Illés et al. 2004; Miyazaki et al. 2011; Willing et al. 2014). Our present finding that FTY720 induced depletion of CD8^hi^ MAITs revealed that CD8 MAITs constituted CD8^hi^ and DN cells in HDs and in MS. Furthermore, depletion of CD8^hi^ MAITs in untreated- and FTY720-treated subjects indicated that this phenomenon was specific for MS irrespective of drug use, suggesting that the phenotype of MAITs was affected in the disease, and the drugs could not restore the original state. Accordingly, the expression of CCR5, an inflammatory chemokine receptor, remained higher in untreated- and FTY720-treated subjects than that in HD CD8^hi^ MAITs, indicating that MAITs were activated (Additional file 5: Figure S3A). Of note, a higher serum level of CCR5 ligands such as CCL3 and CCL5 has been found in MS patients, although the effects of these ligands on the disease has yet to be elucidated (Khaiboullina et al. 2015). Similarly, an increase in serum CCL20, a ligand for CCR6, has been reported in MS patients (Jafarzadeh et al. 2014). The beneficial effects of FTY720 consisted of trapping naive and central memory T and B cells that express CCR7 in lymph nodes, thus preventing egress of the pathogenic cells from recirculation (Brinkmann 2009). A significant decrease in CD4 and CD8 T cells, as well as MAITs, upon FTY720 administration, as shown in Fig. 4, suggested that CCR7^+^ MAITs could be present in MS, probably during the acute relapse stage or in the secondary lymphoid organs, which we did not examine in this study. Given that MAITs exhibit an effector/memory phenotype being CCR7^−^CD28^+^CD27^+^CR45RO^+^ in healthy individuals, such an aberrant phenotype could represent the pathogenic cells (Dusseaux et al. 2011). In line with the hypothesis, an increase in the expression of CD45RA in FTY-2y subjects relative to that in HDs, has been observed, implying that MAITs have become so-called T effector-memory RA (T_EMRA_) cells (Additional file 6: Figure S4B). Because CD8 T_EMRA_ cells stem from central memory T cells (CCR7^+^CD45RA^−^) upon homeostatic proliferation independent of a specific antigen stimulus, the origin of CD45RA^+^ MAITs may be CCR7^+^ cells as described above, provided that the differentiation of MAITs operated similar that of CD8 T_EMRA_ cells (Geginat et al. 2003). Although a recent report has indicated that MAITs are largely CCR7^−^ in untreated MS patients, CCR7^+^ MAITs could be present in FTY720-treated patients (Salou et al. 2016). Furthermore, the enhanced expression of CD107a concomitant with a decline in CD27 after 1 year of FTY 720 therapy indicated that DN MAITs had become terminally differentiated effector/memory cells, although the data for CD57, a marker of terminal differentiation, together with those for perforin, an effector molecule for exerting cytotoxicity, were missing (Fig. 3) (Brenchley et al. 2003). Nonetheless, of note was that this phenotype seemed to be reversed after 2 years of FTY720 treatment, indicating that less mature effector/memory MAITs re-accumulate in the blood (Fig. 3). These results reflected the dynamics of MAITs in MS and may mirror the status of MAITs during the course of drug regimens.

CCR6 is expressed in Th17 cells, which have been considered to be pathogenic in MS (Jadidi-Niaragh and Mirshafiey 2011; Rostami and Ciric 2013). FTY720 traps most Th17 cells within lymph nodes, thus preventing further deleterious effects in the CNS. Indeed, many Th17 cells are found in lesions in the brain of MS patients (Tzartos et al. 2008). Intriguingly, the lesions contained as many IL-17-producing CD8 T cells (Tc17 cells) as Th17 cells (Tzartos et al. 2008). Given that no or only very few CD8 T produces IL-17, and that MAITs are present at lesions, as in the case for Th17 and Tc17 cells, CD8 MAITs would be deleterious in MS. The fact that FTY720 enhanced the frequency of cells producing both TNFα and IFNγ, in MAITs, CD8 T cells, and CD4 T cells, while declining that of TNFα single-producer cells in CD4 T cells suggested beneficial effects of FTY720 on the host defense against infection (Fig. 5b). GM-CSF from pathogenic Th cells has been suggested to play a pivotal role in the pathology of MS by instigating myeloid cells to cause CNS damage (Croxford et al. 2015). Our present data, however, did not detect any differences in the production of GM-CSF from different subject groups irrespective of the cell sources (Fig. 5b). This might be due to the artificial stimulus used in the study, and it is conceivable that MAITs from relapsing MS patients could produce more GM-CSF. The presence of such pathogenic MAITs has yet to be explored.

Increases in the relative frequency of MAITs upon FTY 720 therapy could be due to the loss of naïve and central memory cells from the periphery and suggested that MAITs would continue to play a role in host defense against bacterial and/or fungal pathogens in highly lymphopenic conditions, regardless of the fact that absolute lymphocyte numbers declined sharply (Fig. 4). This hypothesis may be underpinned by a phase 3 trial of FTY720, demonstrating that patients receiving FTY720 or placebo had quasi-identical incidences of upper respiratory and urinary infections, but the occurrence of viral infections increased slightly in the former group at up to 2 years (Calabresi et al. 2014). In line with this, MAITs do not recognize viral proteins (Dusseaux et al. 2011). A follow-up study for more than 3 years should be conducted to see whether MAIT cell number still continue to decrease along with FTY 720 therapy, because depletion of MAITs from the peripheral blood would make patients susceptible to infection, in particular, to opportunistic infection as observed in the case of HIV-infected patients with persistently lowered MAITs (Leeansyah et al. 2013; Sandberg et al. 2013). The significant drop of MAIT cell number could be attributed either to the slow cell death caused by FTY720 or to the death caused by the lack of unknown trophic factors for MAITs being stemmed from other lymphocytes or cells. Alternatively, MAITs may be susceptible to death caused by FTY720-mediated activation. Most importantly, the increase in the relative frequency of CD8^hi^ MAITs concomitant with a decline in DN MAITs after 3 years of FTY 720 therapy suggest that the co-receptor composition of MAITs has recovered to normal except for CD4 MAITs, which had remained low (Fig. 2). Such dynamics of MAITs could reflect to those of MS status, although more studies will be warranted in the future.

## Conclusions

FTY 720 therapy enhanced the relative frequency of MAITs in MS patients in a time-dependent manner. Although the expression of CD8 in MAITs has been affected early by FTY720, longer treatment has reversed the phenotypic change. These data demonstrated that FTY720 induced dynamic change in the relative frequency and in the phenotype of MAITs in MS.

## Additional files

10.1186/s40064-016-2923-9 Availability of the samples during FTY720 treatment. Available samples from the indicated individuals are listed. MAIT: samples available for the analysis of MAIT cell frequency at the indicated time points, surface: samples available for the analysis of the cell surface antigens in MAIT cells at the indicated time points. na: not available.
10.1186/s40064-016-2923-9. List of antibodies used in this study. Antibodies are categorized and the name of the clone is indicated.
10.1186/s40064-016-2923-9. Effects of the disease and drugs on the frequency of CD4 and CD8 T cells. Upper panel; The frequency of CD4 T cells relative to the total CD3^+^ cells in healthy donors (HDs) (n = 10) and disease-modifying treatment free (untreated: UT) (n = 15), and FTY720-treated subjects (n = 7) are plotted. Lower panel; The frequency of CD8 T cells relative to the total CD3^+^ cells in HD (n = 10), untreated (n = 15), and FTY720-treated subjects (n = 7) are plotted. 3 m, 1 y, 2 y, and 3 y; subjects treated FTY720 for 3 months, 1, 2 and 3 years, respectively. Some samples are missing as described in the Materials and Methods. Data are analyzed with Mann–Whitney *U* test (HD vs UT and UT vs IFNβ) or with Kruskal–Wallis test with Dunn’s multiple comparison test for all the possible combinations (among UT, 3 m, 1 y, 2 y, and 3 y). Asterisks indicate the groups showing a significant difference (*: *P* < 0.05, **: *P* < 0.01, ***: *P* < 0.001). ns; not significant.
10.1186/s40064-016-2923-9. Cell surface antigen expression in MAITs from healthy donors. (A) Cell surface antigens exhibiting different levels of expression in DN MAITs and CD8^hi^ MAITs from HDs. (B) Cell surface antigens exhibiting little difference in expression in DN MAITs and CD8^hi^ MAITs from HDs. (A and B) MFI for the indicated cell surface antigens in DN MAITs and CD8^hi^ MAITs from HDs (n = 16) is shown. Asterisks indicate a statistically significant difference (*: *P* < 0.05, **: *P* < 0.01, ***: *P* < 0.001, Mann–Whitney *U* test). (C) Cell surface antigens exhibiting different levels of expression in DN MAITs, CD8^hi^ MAITs and CD4 MAITs from HDs. (D) Cell surface antigens exhibiting little difference in expression in DN MAITs, CD8^hi^ MAITs and CD4 MAITs from HDs. (C and D) The MFI for the indicated cell surface antigens in DN MAITs, CD8^hi^ MAITs, and CD4 MAITs from HDs (n = 16) is shown. Data are analyzed with Kruskal–Wallis test with Dunn’s multiple comparison test for all the possible combinations. Groups showing a difference are indicated with an asterisk (*: *P* < 0.05, **: *P* < 0.01, ***: *P* < 0.001, ****:*P* < 0.0001). All data are presented as horizontal lines: median; boxes: 25th percentile and 75th percentile; whiskers: minimum and maximum.
10.1186/s40064-016-2923-9. Cell surface antigen expression in MAITs in the disease. a Comparative analysis of the cell surface antigen expression in MAITs between HDs and untreated subjects. The MFI for the indicated cell surface antigens in DN MAITs and in CD8^hi^ MAITs from HDs (n = 16) and untreated subjects (n = 15, UT) are plotted. (B) Comparative analysis of cell surface antigen expression in MAITs between untreated and IFNβ-treated subjects. The MFI for the indicated cell surface antigens in DN MAITs and CD8^hi^ MAITs from untreated (n = 15: UT) and IFNβ-treated (n = 14) subjects are plotted. (A and B) *P* < 0.05 indicates a statistical significance (Mann–Whitney U-test). Data are presented as horizontal lines: median; boxes: 25th percentile and 75th percentile; whiskers: minimum and maximum.
10.1186/s40064-016-2923-9. Effects of FTY720 on expression of the cell surface antigens in MAITs. (A) The MFI for the indicated cell surface antigens in DN MAITs and in CD8^hi^ MAITs from untreated (n = 15, UT) subjects and from FTY720-treated patients for 3 months (n = 5, 3 m), 1 year (n = 6, 1 y), and 2 years (n = 5, 2 y) are plotted. Data are analyzed with Kruskal–Wallis test with Dunn’s multiple comparison test for all the possible combinations. (B) Effects of FTY720 on CD45RO and CD45RA MFI for CD45RO and CD45RA in DN MAITs and CD8^hi^ MAITs from HDs (n = 16) and from the FTY720-treated subjects is shown. 3 m, 1 y, and 2 y; subjects treated with FTY720 for 3 months (n = 5), 1 year (n = 6), and 2 years (n = 5), respectively. Data are analyzed with Kruskal–Wallis test with Dunn’s multiple comparison test for all the possible combinations. Asterisks indicate the groups showing a significant difference (*: *P* < 0.05). Data are presented as median. Horizontal line: Median; boxes: 25th percentile and 75th percentile; whiskers: minimum and maximum.
10.1186/s40064-016-2923-9. Gating strategy of intracellular cytokine staining. Left panels: Gating for MAIT cells, CD4 T cells and CD8 T cells is depicted. Production of the indicated cytokines from these cells is measured after permeabilization. The numbers in the figure represent the percentage of the cell populations producing the indicated cytokines. Isotype control staining is also shown for each subset. Representative data are shown.

## Abbreviations

MS: Multiple sclerosis
CNS: Central nervous system
S1P: Sphingisine-1-phosphate
MAITs: Mucosal-Associated Invariant T cells
HDs: Healthy donors
IFN: Interferon
EDSS: Expanded Disability Status Scale
PBMC: Peripheral blood mononuclear cell
FACS: Fluorescence-activated cell sorting
TNF: Tumor necrosis factor
GM-CSF: Granulocyte/macrophage-colony stimulating factor
DN: Double negative
SLAM: Signaling lymphocyte associated molecule

## References

[CR1] Annibali V, Ristori G, Angelini DF, Serafini B, Mechelli R, Cannoni S, Romano S, Paolillo A, Abderrahim H, Diamantini A, Borsellino G, Aloisi F, Battistini L, Salvetti M (2011). CD161(high)CD8+ T cells bear pathogenetic potential in multiple sclerosis. Brain.

[CR2] Birkinshaw RW, Kjer-Nielsen L, Eckle SBG, McCluskey J, Rossjohn J (2014). MAITs, MR1 and vitamin B metabolites. Curr Opin Immunol.

[CR3] Brenchley JM, Karandikar NJ, Betts MR, Ambrozak DR, Hill BJ, Crotty LE, Casazza JP, Kuruppu J, Migueles SA, Connors M, Roederer M, Douek DC, Koup RA (2003). Expression of CD57 defines replicative senescence and antigen-induced apoptotic death of CD8+ T cells. Blood.

[CR4] Brinkmann V (2009). FTY720 (fingolimod) in multiple sclerosis: therapeutic effects in the immune and the central nervous system. Br J Pharmacol.

[CR5] Calabresi PA, Radue E-W, Goodin D, Jeffery D, Rammohan KW, Reder AT, Vollmer T, Agius MA, Kappos L, Stites T, Li B, Cappiello L, von Rosenstiel P, Lublin FD (2014). Safety and efficacy of fingolimod in patients with relapsing-remitting multiple sclerosis (FREEDOMS II): a double-blind, randomised, placebo-controlled, phase 3 trial. Lancet Neurol.

[CR6] Cho Y-N, Kee S-J, Kim T-J, Jin HM, Kim M-J, Jung H-J, Park K-J, Lee S-J, Lee S-S, Kwon Y-S, Kee HJ, Kim N, Park Y-W (2014). Mucosal-associated invariant T cell deficiency in systemic lupus erythematosus. J Immunol.

[CR7] Compston A, Coles A (2008). Multiple sclerosis. Lancet.

[CR8] Confavreux C, Vukusic S (2006). Age at disability milestones in multiple sclerosis. Brain.

[CR9] Croxford AL, Spath S, Becher B (2015). GM-CSF in neuroinflammation: licensing myeloid cells for tissue damage. Trends Immunol.

[CR10] Cui Y, Franciszkiewicz K, Mburu YK, Mondot S, Le Bourhis L, Premel V, Martin E, Kachaner A, Duban L, Ingersoll MA, Rabot S, Jaubert J, De Villartay J-P, Soudais C, Lantz O (2015). Mucosal-associated invariant T cell-rich congenic mouse strain allows functional evaluation. J Clin Invest.

[CR11] Dusseaux M, Martin E, Serriari N, Péguillet I, Premel V, Louis D, Milder M, Le Bourhis L, Soudais C, Treiner E, Lantz O (2011). Human MAIT cells are xenobiotic-resistant, tissue-targeted, CD161hi IL-17-secreting T cells. Blood.

[CR12] Geginat J, Lanzavecchia A, Sallusto F (2003). Proliferation and differentiation potential of human CD8 + memory T-cell subsets in response to antigen or homeostatic cytokines. Blood.

[CR13] Huseby ES, Huseby PG, Shah S, Smith R, Stadinski BD (2012). Pathogenic CD8 T cells in multiple sclerosis and its experimental models. Front Immunol.

[CR14] Illés Z, Shimamura M, Newcombe J, Oka N, Yamamura T (2004). Accumulation of Valpha7.2-Jalpha33 invariant T cells in human autoimmune inflammatory lesions in the nervous system. Int Immunol.

[CR15] Jadidi-Niaragh F, Mirshafiey A (2011). Th17 cell, the new player of neuroinflammatory process in multiple sclerosis. Scand J Immunol.

[CR16] Jafarzadeh A, Bagherzadeh S, Ebrahimi HA, Hajghani H, Bazrafshani MR, Khosravimashizi A, Nemati M, Gadari F, Sabahi A, Iranmanesh F, Mohammadi MM, Daneshvar H (2014). Higher circulating levels of chemokine CCL20 in patients with multiple sclerosis: evaluation of the influences of chemokine gene polymorphism, gender, treatment and disease pattern. J Mol Neurosci.

[CR17] Khaiboullina SF, Gumerova AR, Khafizova IF, Martynova EV, Lombardi VC, Bellusci S, Rizvanov AA (2015). CCL27: novel cytokine with potential role in pathogenesis of multiple sclerosis. Biomed Res Int.

[CR18] Le Bourhis L, Guerri L, Dusseaux M, Martin E, Soudais C, Lantz O (2011). Mucosal-associated invariant T cells: unconventional development and function. Trends Immunol.

[CR19] Leeansyah E, Ganesh A, Quigley MF, Sönnerborg A, Andersson J, Hunt PW, Somsouk M, Deeks SG, Martin JN, Moll M, Shacklett BL, Sandberg JK (2013). Activation, exhaustion, and persistent decline of the antimicrobial MR1-restricted MAIT-cell population in chronic HIV-1 infection. Blood.

[CR20] Lim SY, Constantinescu CS (2010). Current and future disease-modifying therapies in multiple sclerosis. Int J Clin Pract.

[CR21] Miyazaki Y, Miyake S, Chiba A, Lantz O, Yamamura T (2011). Mucosal-associated invariant T cells regulate Th1 response in multiple sclerosis. Int Immunol.

[CR22] Polman CH, Reingold SC, Banwell B, Clanet M, Cohen JA, Filippi M, Fujihara K, Havrdova E, Hutchinson M, Kappos L, Lublin FD, Montalban X, O’Connor P, Sandberg-Wollheim M, Thompson AJ, Waubant E, Weinshenker B, Wolinsky JS (2011). Diagnostic criteria for multiple sclerosis: 2010 revisions to the McDonald criteria. Ann Neurol.

[CR23] Pröbstel A-K, Sanderson NSR, Derfuss T (2015). B cells and autoantibodies in multiple sclerosis. Int J Mol Sci.

[CR24] Rahimpour A, Koay HF, Enders A, Clanchy R, Eckle SBG, Meehan B, Chen Z, Whittle B, Liu L, Fairlie DP, Goodnow CC, McCluskey J, Rossjohn J, Uldrich AP, Pellicci DG, Godfrey DI (2015). Identification of phenotypically and functionally heterogeneous mouse mucosal-associated invariant T cells using MR1 tetramers. J Exp Med.

[CR25] Roederer M, Nozzi JL, Nason MC (2011). SPICE: exploration and analysis of post-cytometric complex multivariate datasets. Cytometry A.

[CR26] Rostami A, Ciric B (2013). Role of Th17 cells in the pathogenesis of CNS inflammatory demyelination. J Neurol Sci.

[CR27] Salou M, Nicol B, Garcia A, Baron D, Michel L, Elong-Ngono A, Hulin P, Nedellec S, Jacq-Foucher M, Le Frère F, Jousset N, Bourreille A, Wiertlewski S, Soulillou JP, Brouard S, Nicot AB, Degauque N, Laplaud DA (2016). Neuropathologic, phenotypic and functional analyses of Mucosal Associated Invariant T cells in Multiple Sclerosis. Clin Immunol.

[CR28] Sandberg JK, Dias J, Shacklett BL, Leeansyah E (2013). Will loss of your MAITs weaken your HAART?. AIDS.

[CR29] Serriari N-E, Eoche M, Lamotte L, Lion J, Fumery M, Marcelo P, Chatelain D, Barre A, Nguyen-Khac E, Lantz O, Dupas J-L, Treiner E (2014). Innate mucosal-associated invariant T (MAIT) cells are activated in inflammatory bowel diseases. Clin Exp Immunol.

[CR30] Song Z-Y, Yamasaki R, Kawano Y, Sato S, Masaki K, Yoshimura S, Matsuse D, Murai H, Matsushita T, Kira J-I (2014). Peripheral blood T cell dynamics predict relapse in multiple sclerosis patients on fingolimod. PLoS ONE.

[CR31] Sugimoto C, Konno T, Wakao R, Fujita H, Fujita H, Wakao H (2015). Mucosal-associated invariant T cell is a potential marker to distinguish fibromyalgia syndrome from arthritis. PLoS ONE.

[CR32] Teunissen MBM, Yeremenko NG, Baeten DLP, Chielie S, Spuls PI, de Rie MA, Lantz O, Res PCM (2014). The IL-17A-producing CD8+ T-cell population in psoriatic lesional skin comprises mucosa-associated invariant T cells and conventional T cells. J Invest Dermatol.

[CR33] Treiner E, Liblau RS (2015). Mucosal-associated invariant T cells in multiple sclerosis: the Jury is still out. Front Immunol.

[CR34] Tzartos JS, Friese MA, Craner MJ, Palace J, Newcombe J, Esiri MM, Fugger L (2008). Interleukin-17 production in central nervous system-infiltrating T cells and glial cells is associated with active disease in multiple sclerosis. Am J Pathol.

[CR35] Wakao H, Yoshikiyo K, Koshimizu U, Furukawa T, Enomoto K, Matsunaga T, Tanaka T, Yasutomi Y, Yamada T, Minakami H, Tanaka J, Oda A, Sasaki T, Wakao R, Lantz O, Udagawa T, Sekiya Y, Higuchi K, Harada N, Nishimura K, Ohtaka M, Nakanishi M, Fujita H (2013). Expansion of functional human mucosal-associated invariant T cells via reprogramming to pluripotency and redifferentiation. Cell Stem Cell.

[CR36] Willing A, Leach OA, Ufer F, Attfield KE, Steinbach K, Kursawe N, Piedavent M, Friese MA (2014). CD8^+^ MAIT cells infiltrate into the CNS and alterations in their blood frequencies correlate with IL-18 serum levels in multiple sclerosis. Eur J Immunol.

