# Supplementary figures and images for: The dynamics of mucosal-associated invariant T cells in multiple sclerosis
Source: Springerplus. 2016 Aug 5;5(1):1259. doi: 10.1186/s40064-016-2923-9 (PMC4974206; doi:10.1186/s40064-016-2923-9)

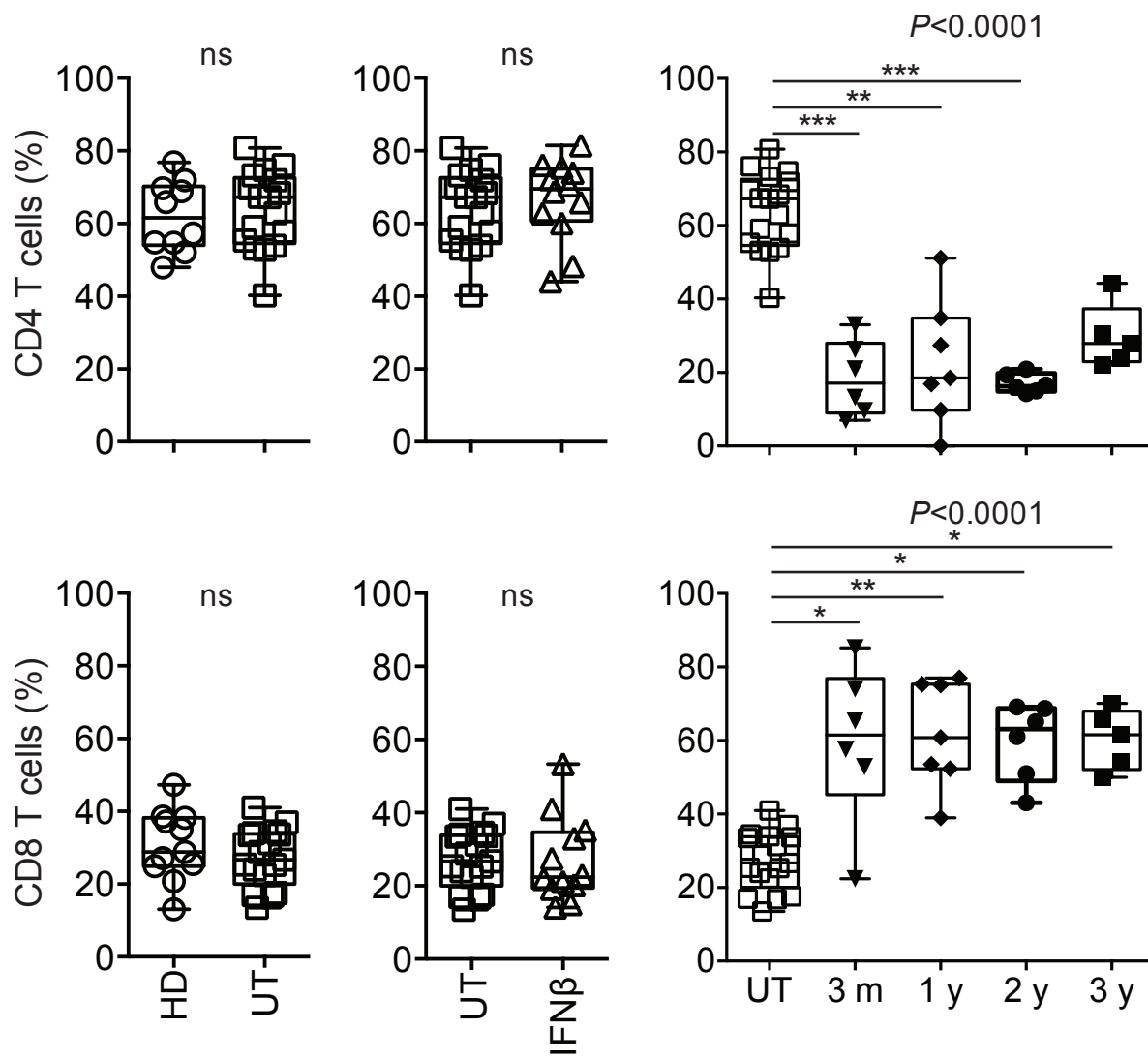

Supplement: Supplementary file 3 — 10.1186/s40064-016-2923-9. Effects of the disease and drugs on the frequency of CD4 and CD8 T cells. Upper panel; The frequency of CD4 T cells relative to the total CD3+ cells in healthy donors (HDs) (n = 10) and disease-modifying treatment free (untreated: UT) (n = 15), and FTY720-treated subjects (n = 7) are plotted. Lower panel; The frequency of CD8 T cells relative to the total CD3+ cells in HD (n = 10), untreated (n = 15), and FTY720-treated subjects (n = 7) are plotted. 3 m, 1 y, 2 y, and 3 y; subjects treated FTY720 for 3 months, 1, 2 and 3 years, respectively. Some samples are missing as described in the Materials and Methods. Data are analyzed with Mann–Whitney U test (HD vs UT and UT vs IFNβ) or with Kruskal–Wallis test with Dunn’s multiple comparison test for all the possible combinations (among UT, 3 m, 1 y, 2 y, and 3 y). Asterisks indicate the groups showing a significant difference (*: P < 0.05, **: P < 0.01, ***: P < 0.001). ns; not significant. [file 40064_2016_2923_MOESM3_ESM.pdf]

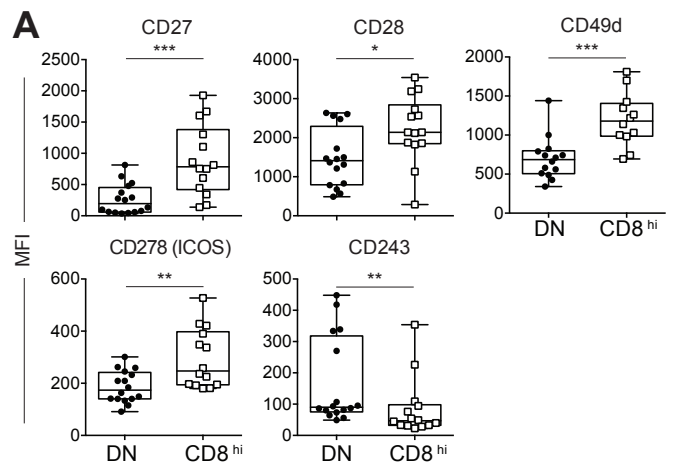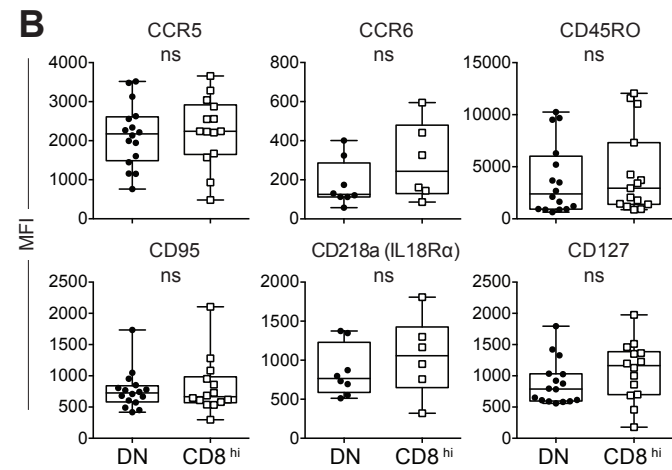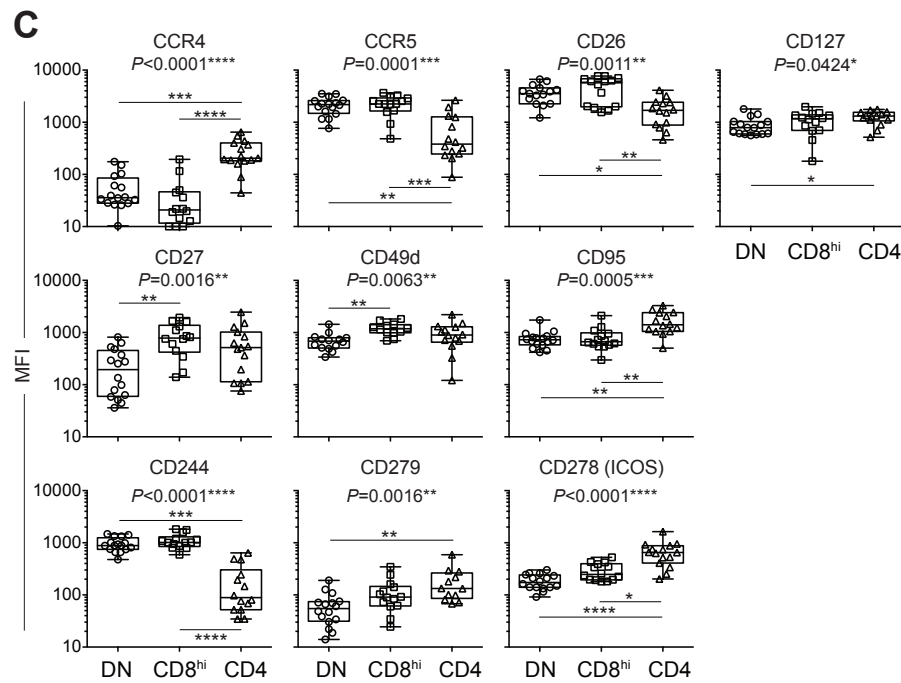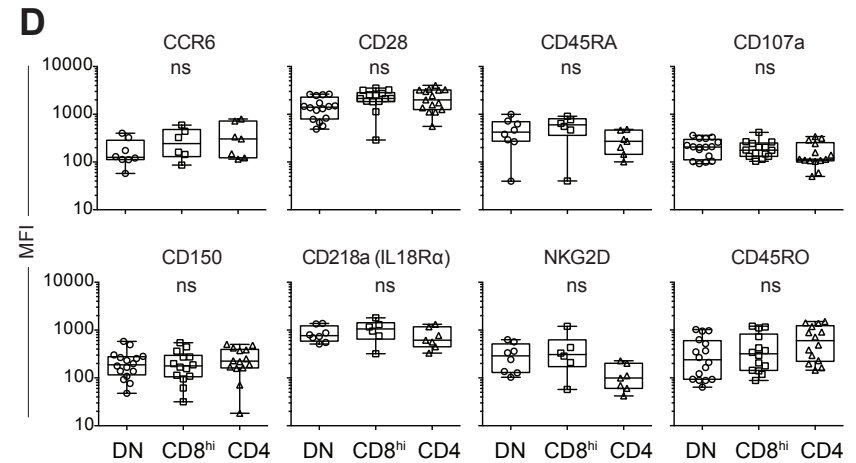

Supplement: Supplementary file 4 — 10.1186/s40064-016-2923-9. Cell surface antigen expression in MAITs from healthy donors. (A) Cell surface antigens exhibiting different levels of expression in DN MAITs and CD8hi MAITs from HDs. (B) Cell surface antigens exhibiting little difference in expression in DN MAITs and CD8hi MAITs from HDs. (A and B) MFI for the indicated cell surface antigens in DN MAITs and CD8hi MAITs from HDs (n = 16) is shown. Asterisks indicate a statistically significant difference (*: P < 0.05, **: P < 0.01, ***: P < 0.001, Mann–Whitney U test). (C) Cell surface antigens exhibiting different levels of expression in DN MAITs, CD8hi MAITs and CD4 MAITs from HDs. (D) Cell surface antigens exhibiting little difference in expression in DN MAITs, CD8hi MAITs and CD4 MAITs from HDs. (C and D) The MFI for the indicated cell surface antigens in DN MAITs, CD8hi MAITs, and CD4 MAITs from HDs (n = 16) is shown. Data are analyzed with Kruskal–Wallis test with Dunn’s multiple comparison test for all the possible combinations. Groups showing a difference are indicated with an asterisk (*: P < 0.05, **: P < 0.01, ***: P < 0.001, ****:P < 0.0001). All data are presented as horizontal lines: median; boxes: 25th percentile and 75th percentile; whiskers: minimum and maximum. [file 40064_2016_2923_MOESM4_ESM.pdf]

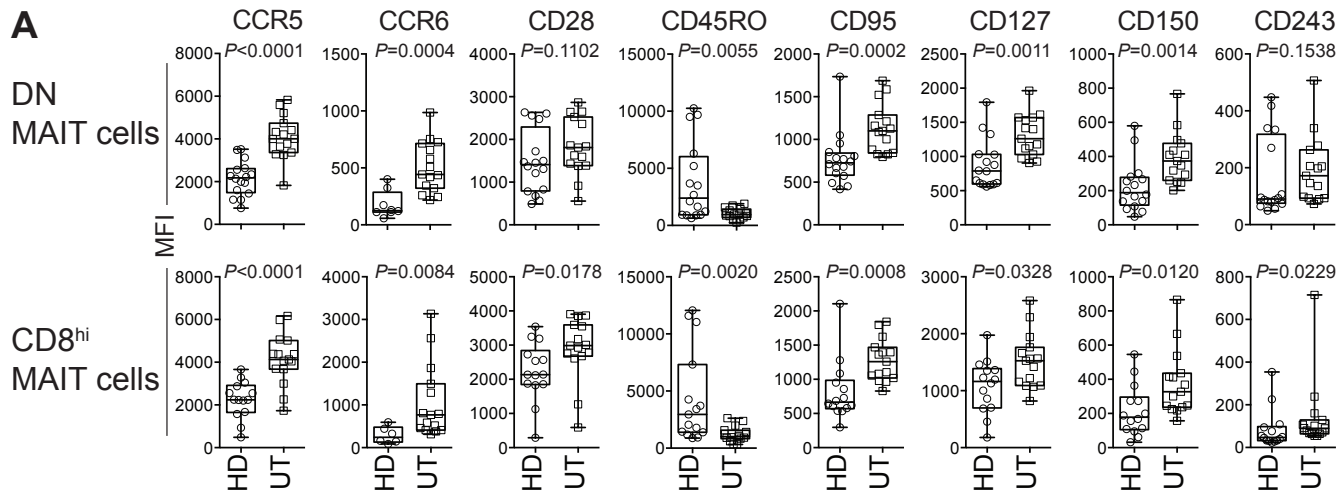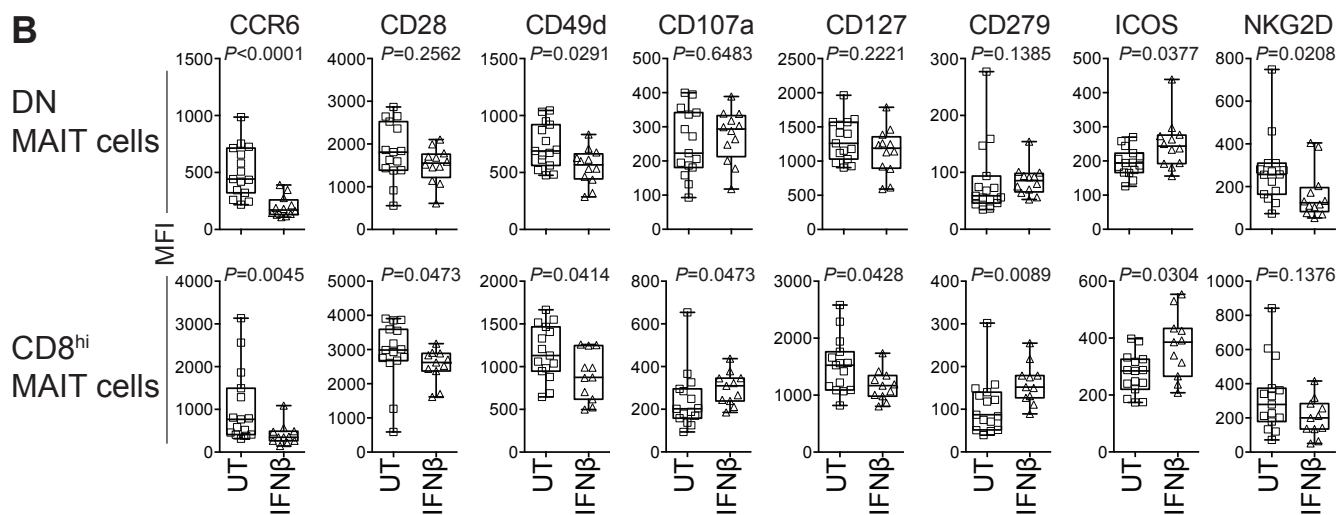

Supplement: Supplementary file 5 — 10.1186/s40064-016-2923-9. Cell surface antigen expression in MAITs in the disease. a Comparative analysis of the cell surface antigen expression in MAITs between HDs and untreated subjects. The MFI for the indicated cell surface antigens in DN MAITs and in CD8hi MAITs from HDs (n = 16) and untreated subjects (n = 15, UT) are plotted. (B) Comparative analysis of cell surface antigen expression in MAITs between untreated and IFNβ-treated subjects. The MFI for the indicated cell surface antigens in DN MAITs and CD8hi MAITs from untreated (n = 15: UT) and IFNβ-treated (n = 14) subjects are plotted. (A and B) P < 0.05 indicates a statistical significance (Mann–Whitney U-test). Data are presented as horizontal lines: median; boxes: 25th percentile and 75th percentile; whiskers: minimum and maximum. [file 40064_2016_2923_MOESM5_ESM.pdf]

**A**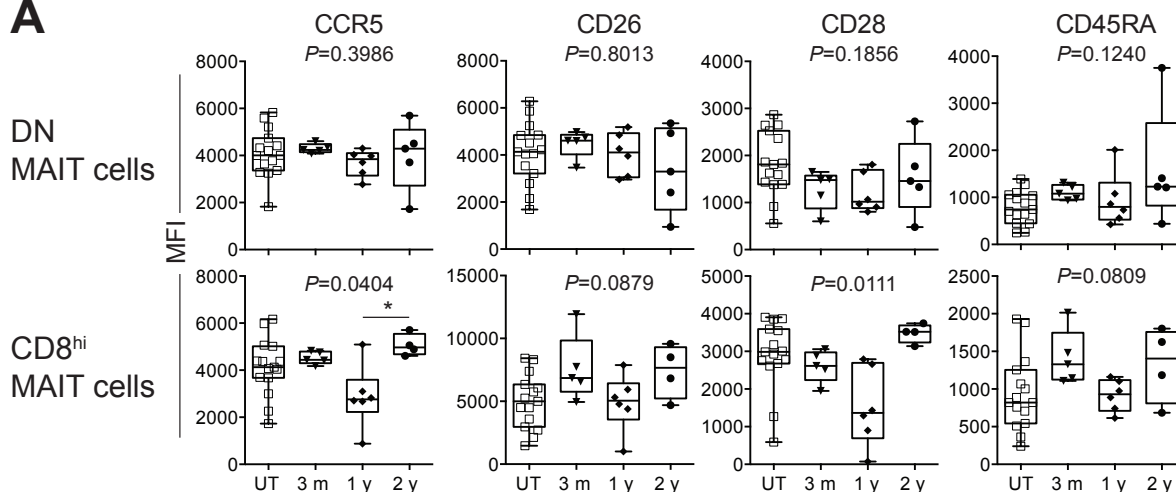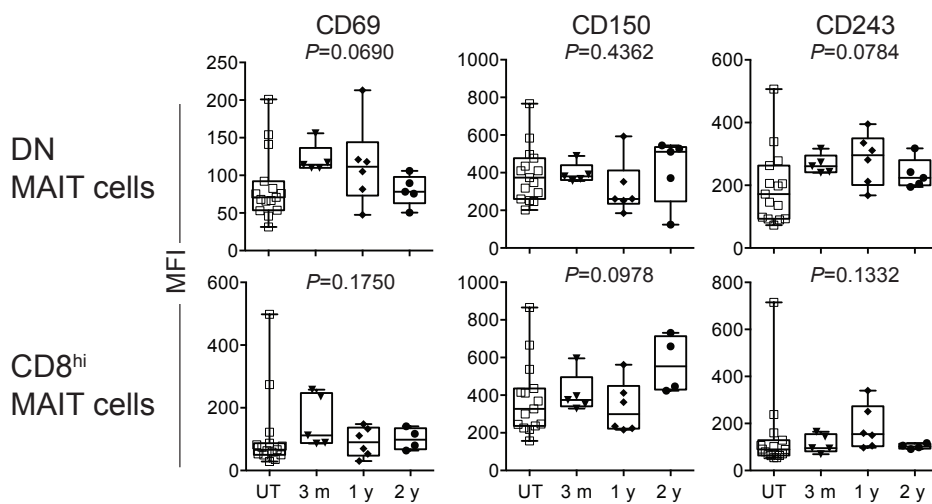**B**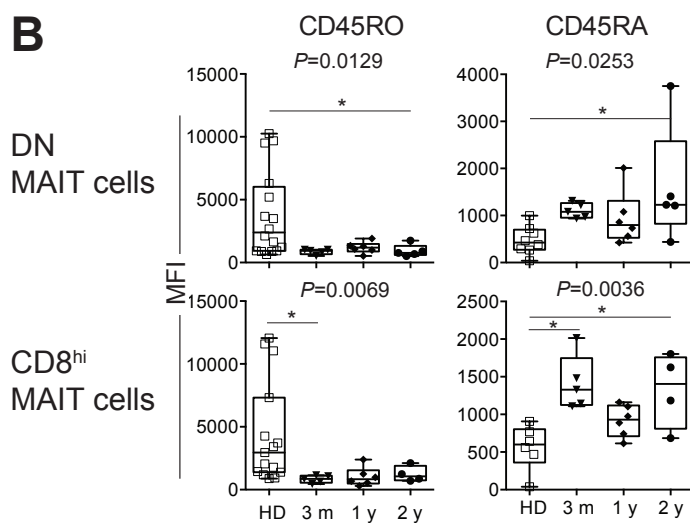

Supplement: Supplementary file 6 — 10.1186/s40064-016-2923-9. Effects of FTY720 on expression of the cell surface antigens in MAITs. (A) The MFI for the indicated cell surface antigens in DN MAITs and in CD8hi MAITs from untreated (n = 15, UT) subjects and from FTY720-treated patients for 3 months (n = 5, 3 m), 1 year (n = 6, 1 y), and 2 years (n = 5, 2 y) are plotted. Data are analyzed with Kruskal–Wallis test with Dunn’s multiple comparison test for all the possible combinations. (B) Effects of FTY720 on CD45RO and CD45RA MFI for CD45RO and CD45RA in DN MAITs and CD8hi MAITs from HDs (n = 16) and from the FTY720-treated subjects is shown. 3 m, 1 y, and 2 y; subjects treated with FTY720 for 3 months (n = 5), 1 year (n = 6), and 2 years (n = 5), respectively. Data are analyzed with Kruskal–Wallis test with Dunn’s multiple comparison test for all the possible combinations. Asterisks indicate the groups showing a significant difference (*: P < 0.05). Data are presented as median. Horizontal line: Median; boxes: 25th percentile and 75th percentile; whiskers: minimum and maximum. [file 40064_2016_2923_MOESM6_ESM.pdf]

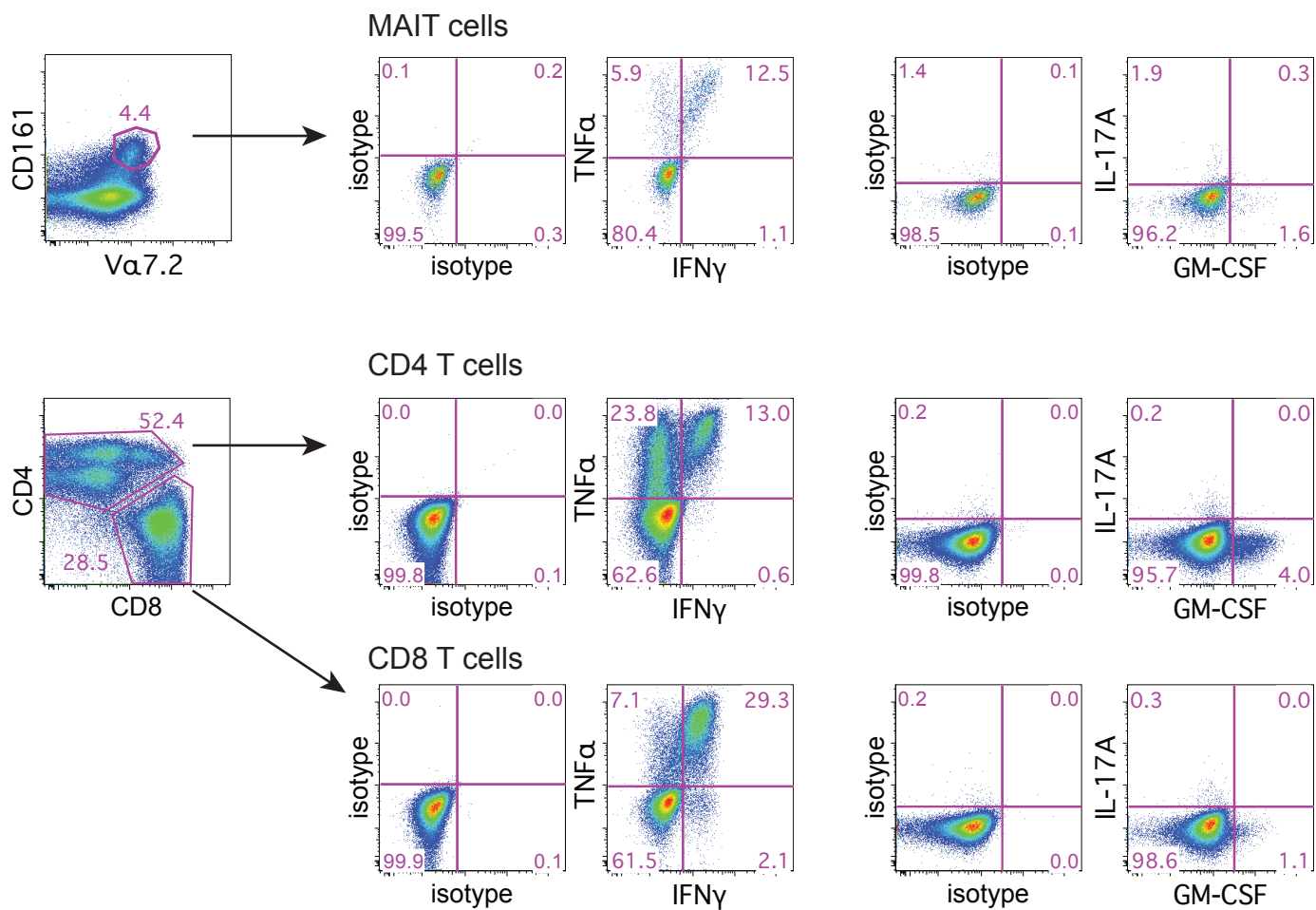

Supplement: Supplementary file 7 — 10.1186/s40064-016-2923-9. Gating strategy of intracellular cytokine staining. Left panels: Gating for MAIT cells, CD4 T cells and CD8 T cells is depicted. Production of the indicated cytokines from these cells is measured after permeabilization. The numbers in the figure represent the percentage of the cell populations producing the indicated cytokines. Isotype control staining is also shown for each subset. Representative data are shown. [file 40064_2016_2923_MOESM7_ESM.pdf]
